# Retrospective surveillance of severe acute respiratory syndrome coronavirus 2 in pets from Brazil

**DOI:** 10.14202/vetworld.2021.2803-2808

**Published:** 2021-10-28

**Authors:** Otávio Valério de Carvalho, Luiz Eduardo Ristow, Davi dos Santos Rodrigues, Cláudia Kathariny da Silva Farias, Rita de Cássia Carvalho Maia

**Affiliations:** 1 TECSA Laboratories, Av. do Contorno, 6226 - Funcionários, Belo Horizonte - MG, 30110-042, Brazil; 2 Department of Veterinary Medicine, LAVIAN, Federal Rural University of Pernambuco, Dom Manoel de Medeiros Street, S/N, Recife-PE, 52171-900, Brazil.

**Keywords:** companion animals, coronavirus disease-19, vigilance, virus

## Abstract

**Background and Aim:**

The emerging concerns regarding the new Coronavirus’s ability to cause infection in pets has led to animal testing and worrisome findings reported all over the world in domesticated and wild animals. This study aimed to investigate severe acute respiratory syndrome coronavirus (SARS-CoV)-2 by quantitative reverse transcription-polymerase chain reaction in dog and cat samples with the clinical presentation for respiratory or gastrointestinal disease in Brazil.

**Materials and Methods:**

One hundred and twenty-five samples were collected from 12 states of Brazil that originated from the gastrointestinal, upper respiratory tract, and other sites, including some pools of samples from before the onset of the pandemic including blood and/or urine samples. They were tested for RT-PCR detection of respiratory or gastrointestinal pathogens through Respiratory or Diarrhea RT-PCR Panels in the TECSA (Tecnologia em Saninade Animal - Animal Health Technology) Veterinary Medicine Laboratory. This work was conducted in compliance with ethical standards.

**Results:**

Seven different microorganisms that can cause respiratory and/or gastrointestinal clinical signs were detected in cats (Feline Coronavirus [FCoV], Feline Parvovirus, Feline Leukemia Virus, Feline Calicivirus, *Mycoplasma felis*, *Campylobacter* spp., and *Cryptosporidium* spp.) and three in dogs (canine distemper virus, *Cryptosporidium* spp., and *Babesia* spp.).

**Conclusion:**

Although the samples corresponded to the beginning of coronavirus disease-19 spread in Brazil and clinically correlated with the expected viral replication sites, none of the animals tested positive for SARS-CoV-2; reassuringly, four cats tested positive or FCoV none of them were positive for SARS-CoV2. The epidemiological surveillance of SARS-CoV-2 in pets is considered a one health issue, important for monitoring the disease evolution, spread and minimizing the animal-human health impacts, and directing Public Health Policies.

## Introduction

The emerging concerns regarding the new Coronavirus’ (severe acute respiratory syndrome coronavirus [SARS-CoV-2]) ability to cause infection in pets, considering its animal origin [1], has led to animal testing and worrisome findings reported all over the world in different kinds of domesticated animals [2]. From this point, SARS-CoV-2 has been considered an anthropozoonosis and one health approach may be considered, ultimately inferring that those humans infected with the virus should restrain contact with domestic animals [3,4].

With the total count of SARS-CoV-2 in the world exponentially increasing, some countries question whether the infection started before the onset of the first human case officially reported [5,6]. It also has been discussed that pets or companion animals have been a source of constant worry and testing for virus and antibody detection surveillances has started on them [2]. In Brazil, the first official SARS-CoV-2 human case was reported on February 26, 2020 [7] and the cases have been increasing along with the high number of death cases, which may raise the question of whether the human infection could have arisen earlier in the country and whether companion animals may serve as an indicator of human cases.

In August 2021, Brazil has reached 20,528,099 confirmed cases and 573,511 confirmed deaths from coronavirus disease -19 (COVID-19) [8]. Therefore, considering its large population, also the fact that is one of the latest big countries to report a human case, and to survey the possibility of infection by SARS-CoV-2 in Brazil happening before that first official human case, we accessed biological samples from animals presenting symptoms all over Brazil from February to April 2020.

## Materials and Methods

### Ethical approval

The clinical samples submitted to this investigation were collected for the purpose of routine health monitoring by veterinary clinics. This study did not involve the handling of live or dead animals. According to the applicable legislation (Ethics Committee on Animals Use of Federal Rural University of Pernambuco), ethics approval was not required for this study.

### Study period and location

The study was conducted from February to April 2020 at TECSA Laboratories, Belo Horizonte, Minas Gerais, Brazil.

### Animals and experimental protocol

Clinical samples of 69 dogs and 56 cats (total of 125 samples) were from both sexes and different breeds and ages. The samples came from animals from all over Brazil to be tested for different diseases at TECSA Laboratories.

The panel of tests performed was grouped by known disease characteristics, such as gastrointestinal problems or respiratory tract-related problems. Those animals were evaluated by veterinary physicians and samples were also submitted by them. Information on the household’s details is not available for disclosure. Since the animals showed symptoms accordingly, samples were tested initially for real-time polymerase chain reaction (RT-PCR) detection of respiratory or gastrointestinal pathogens through Respiratory or Diarrhea RT-PCR Panels from TECSA Laboratories. These diagnostic panels offered by the laboratory allow to screen for the most common infectious causes of canine/feline respiratory or gastrointestinal disorders (Table-1), symptoms lately associated with SARS-CoV2 virus infection site in animals [2,9]. Most samples were collected from the upper respiratory tract (nasal, ocular, and/or oropharyngeal swab), followed by gastrointestinal samples (feces or rectal swab) and others, including some pools of the previous samples (blood and/or urine).

**Table-1:** Data from analyzed samples along with SARS-CoV-2 testing.

Animal	Number of animals	Sampling date			RT-qPCR SARS-CoV-2 (number of positive animals)			Other pathogens detected (number of positive animals)
	
		Feb	Mar	Apr	Target N1	Target N2	Target N3	
Cat	56	1	21	34	0	0	0	FeLV (05) FCoV (04) *Mycoplasma felis* (03) FCV (02) *Campylobacter* spp. (01) *Cryptosporidium* spp. (01) FPV (01)
Dog	69	1	35	33	0	0	0	CDV (30) *Cryptosporidium* spp. (01) *Babesia* spp. (01)
Total	125	2	56	67	0	0	0	Other pathogens (44)

FeLV=Feline immunodeficiency virus, FCoV=Feline coronavirus, FCV=Feline calicivirus, FPV=Feline panleukopenia virus, CDV=Canine distemper virus.

**Figure-1:**
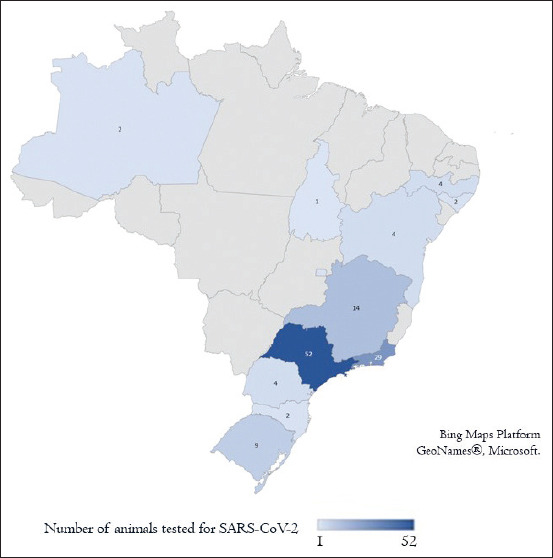
Origin and number of samples tested from each state of Brazil [Source: https://www.geonames.org/v3/].

Samples came from 12 states and represent the five regions of Brazil: São Paulo, Rio de Janeiro, Minas Gerais (southeastern region), Rio Grande do Sul, Paraná, Santa Catarina (southern region), Bahia, Pernambuco, Alagoas (northeastern region), Amazonas (northern region), and Distrito Federal and Tocantins (central-western region) (Figure-1). Sampling was much higher in the Southeastern region of Brazil (76%), mainly from São Paulo state (41.6%). All samples were stored at −80°C until used.

Before RNA isolation, tubes containing swab samples were first embedded in phosphate-buffered saline (pH 7.2) and then vortexed. Swab suspension was transferred to a new test tube. Feces debris was discarded by centrifugation (Novatecnica NT-805, Brazil – Refrigerated Microcentrifuge) at 8,000 xg for 3 minutes at 4°C. RNA was extracted from clinical samples with the Maxwell Rapid Sample Concentrator simplyRNA Tissue kit (Promega^®^, USA) according to the manufacturer’s instructions. SARS-CoV-2 quantitative reverse transcription PCR (RT-qPCR) assays were performed using GoTaq Probe 1-Step RT-qPCR System (Promega) and 2019- novel coronavirus (nCoV) CDC EUA kit (Integrated DNA Technologies, USA) and following the respective manufacturer’s protocols. Three primer-probe sets targeting the nucleocapsid gene (N1, N2, and N3) were determined according to the CDC 2019-nCoV Real-Time RT-PCR Diagnostic Panel [10]. N1 and N2 are specific to SARS-CoV-2 and N3 is specific to SARS-like viruses. Additional primer-probe sets to detect the canine and feline beta-actin (ACTB) housekeeping gene were also included in the assays as endogenous controls. Amplification was carried out in a QuantStudio 1 RT-PCR System (Applied Biosystems^®^, Singapore).

## Results

The ACTB-specific amplification was demonstrated in all assays; however, neither the dogs nor cats have tested positive to SARS-CoV-2 RT-qPCR (Table-1). On the other hand, 30 dogs and 14 cats were positive for other pathogens as differential diagnoses. Seven different microorganisms that can cause respiratory and/or gastrointestinal clinical signs were detected in cats, with five positive for feline leukemia virus, four positive for feline coronavirus (FCoV), three cats were positive for *Mycoplasma felis*, two were positive for feline calicivirus (FCV), one cat was positive for *Campylobacter* spp., one for *Cryptosporidium* spp., and another one was positive for feline parvovirus (FPV).

Among the dogs, three diseases were detected: Three dogs were positive for canine distemper virus (CDV), one for *Cryptosporidium* spp., and only one was positive for *Babesia* spp.). From all samples, coinfections were observed for three cats (FCoV plus FPV, FCV plus *M. felis*, and *Campylobacter* spp. plus *Cryptosporidium* spp.) and for two dogs (CDV plus *Cryptosporidium* spp. and CDV plus *Babesia* spp.). A very reassuring finding was the fact that, although we had four positive cats for FCoV, none of them tested positive for SARS-CoV2, demonstrating that the PCR test does not cross-react among the viruses.

## Discussion

The beginning of SARS-CoV-2 pandemics in November 2019 in China and its further and devastating increase in human population has been raising many questions ranging from its origins to all ­epidemiological aspects of the disease [11]. As considered a new disease, SARS-CoV-2 has been unveiling its pathogenesis gradually and leading scientists to analyze the basis of its One Health intricacy [12]. Some environmental factors may be implicated at the beginning of the SARS-CoV-2 epidemic, such as progressive deforestation, anthropization, and exploration of threatened wildlife.

The assessment of risk factors and health epidemiological surveillance to track the prevalence and circulation of SARS-Cov2 in the wild animal population, especially those living around rural properties, and stray animals, especially felines, are necessary for containment of viral dissemination and propagation and must be constant through the serological investigation of those animals that reside and/or circulate in the vicinity of virus outbreaks. SARS-CoV2 represents a risk to both animals and public health, as it surpasses the species barrier and adapts to evolution, influencing the dynamics of viral transmission. Due to the constant mutation capacity of this zoonotic coronavirus, there is the emergence of new variants of the virus that may pose a threat to vaccine efficacy and diagnostic detection [13-17].

Epidemiological surveillance for animal coronaviruses is a fundamental movement of “One Health,” in the past 20 years, there have been three events of animal CoV spillovers into humans. Strict environmental management policies are essential to prevent the occurrence of future pandemics [18]. The discovery of asymptomatic/presymptomatic carriers demonstrated the potential of the virus to easily spread and develop disease foci impossible to track [6]. Another incredible feature of the disease was the discovery of companion animals in the epidemiology of SARS-CoV-2.

Although the pandemic is extremely recent, from the beginning of 2020, transmission has been observed in dogs and cats, with the first case, a 17 year old Pomeranian Loulou, belonging to a SARS-CoV-2-positive tutor from Hong Kong, showed low levels of virus load in saliva and nasal secretion samples, and later also demonstrated the presence of anti-SARS-CoV-2 antibody. The second case in a dog has been reported where a German Shepherd, from a German resident of Hong Kong, also SARS-CoV-2-positive tutor has tested positive for the virus, although no symptoms were developed and the virus titer was very low. In this case, virus sequences from owner and dog were identical, indicating transmission from owner to pet. The cases involving cats were very similar to those described for dogs; the cats belonged to SARS-CoV-2-positive owners, one from Belgium and one from Hong Kong, and from both animals’ nasal, oral, and rectal samples showed low virus titers and the cats developed no symptoms [4].

Bringing more complexity to the case, Shi *et al*. [2] demonstrated experimentally that cats and ferrets are susceptible to SARS-CoV-2, but poorly replicates in dogs, chickens, ducks, and pigs. The general structures of the receptor-binding domain (RBD) and angiotensin-converting enzyme 2 (dACE2) of dogs are similar to the human RBD/ACE2 complex; however, the interaction of the two complexes is different, justifying the difficulty of viral replication in this species [19].

There are very few reports of symptoms of SARS-CoV-2 in animals, usually they are correlated to virus replication sites [2,11]. In our daily basis contact with veterinarians, we hear that some cats, when in closer contact with SARS-CoV-2-positive humans, may present low level symptoms, such as loss of smell and a low-grade fever. The previous study suggests that infected cats can transmit SARS-CoV-2 to healthy cats [20], although so far, it has not been shown the possibility of transmission from cat to man and considering the feline susceptibility it is recommended preventive measures against exposing cats to SARS-CoV-2-infected persons to avoid viral dissemination [21]. Coronavirus from cats and dogs belong to the alfacoronavirus genre. Meanwhile, SARS-CoV-2, infecting humans, belong to the betacoronavirus, moreover feline and canine belong to distinct clades and are distant from SARS-CoV-2 with nucleotide similarities from 44 to 44.5% [22].

The present study showed that samples from animals with symptoms similar to SARS-CoV-2 from different regions of Brazil (Figure-1), sent to test for other diseases in a clinical laboratory in Brazil, tested negative for the presence of the virus. This is an important finding, considering that in the context of globalization, the development of these pandemics takes a very fast route [23], which allied to underreporting and low testing rates disguise the entry of the virus in the country. Addressing this possibility, this study used previously collected samples in extremely good preservation conditions to try to bring some answers to this question.

Considering in our study, we covered samples from before the first official human positive case of SARS-CoV-2 in Brazil, February 26, and all the way through high levels of populations infections in March and April [5], we were able to survey the presence of the virus in animals with symptoms that could have been overlooked if coming from households where no tests have been performed in humans, either due to low testing protocol in Brazil or coming from asymptomatic people.

Our results showed that none of the samples tested were positive for SARS-CoV-2, and this brings good news regarding the retrospective possible animal cases in Brazil, but also still leaves questions to asymptomatic animals in those households. This question may not be answered, considering that future testing for anti-SARS-CoV-2 antibody in the majority of households pets in Brazil is an unlikely perspective, and also, for loss of the so called window of opportunity with the progression of the disease, many animals may have had infection and present the antibodies, but we still will not know when it started, considering we only have access to samples previous to the onset of the disease in the country from animals with symptoms that needed medical attention and not the asymptomatic ones.

SARS-CoV-2 infection in cats in particular needs better understanding and to be continuously monitored. Although there is no report of transmissibility to humans, these animals might work as a silent intermediate host since infected cats to date do not reproduce a clinical pattern that can be easily recognized as COVID-19 [24]. Another important finding in our work was the observation that although we still had four cats testing positive for FCoV (Table-1), the protocol indicated by the CDC [10] did not detect them, which gives us higher confidence in the capacity of the test to not generate false-positive results among other Coronaviruses species.

Our data show that no dog or cat tested positive for SARS-CoV2 in the evaluated time interval. This result needs to be contextualized, considering a few facts that limit the sensitivity of such a surveillance system. First, to perceive the probability of positive animals among our samples we must consider that an animal being brought to a veterinary clinic and tested for any disease is directly connected with the salience of symptoms, since healthy pets are rarely tested on a schedule – regardless of health status – in Brazil. It has recently been shown that dogs are less susceptible to SARS-CoV2 than cats; infected cats, on the other hand, have reportedly not presented standardized symptoms [25]. This probably indicates that there is no need for perceptible symptoms to imply that an animal has the virus, causing a loop, since, without any sort of symptom, we would not have had the retrospective samples from a clinical laboratory, especially before the implication of animals epidemiologically involved in the disease. Hence, our samples being originated from animals with symptoms limit the access to samples from animals without symptoms.

We also must consider that regular access of animals to veterinary care is strongly influenced by the socioeconomic status of the tutor, which varies significantly among states and regions in Brazil. Our sampling access, therefore, excludes testing a large part of the population with all sorts of symptomatic profiles during that period. Another relevant observation is the closeness of the animal and its tutor that could increase the chance of transmission, in principle. Regions with higher degrees of urbanization have more animals confined in apartments, which augments the likelihood of proximity. Especially in the context of social isolation, contact between humans and pets has become even more frequent. Our results show that most samples (76%) came from the Southeastern region, a region with the higher urbanization level in Brazil [26].

The last point of consideration is the sampling timing since the samples date from a period right before the first confirmed human case in Brazil (February 26) until the end of April 2021, when human cases were nationally already high [1]. Considering that the increase in the number of human cases could also influence the number of animal cases, our data showed a higher number of samples from the state of São Paulo (41.6%) than any other state (Figure-1), this state is localized in the Southeastern region, with the higher urbanization level, and presents the higher number of both human cases and deaths by SARS-CoV2 in Brazil during the testing period, and also it is where the first human case was discovered in Brazil.

Taking together this rationale shows us that testing animals may still be helpful in studying the epidemiology of the disease, as the limitations of accessible samples could be clearly overcome by a one health approach and its systematic interpretation of illness.

## Conclusion

Various mechanisms should be considered to prevent the pandemic from advancing, one of which is interrupting transmission pathways. A very efficient way to evaluate mechanisms to prevent disease advancement is using One Health principles. Considering facts beyond human life, involving disease aspects in animals and the environment can improve prophylaxis and control measures. Our data showed that no animals from our sample bank showed positive for SARS-CoV2 prior and during the early onset of the disease in Brazil, raising the question about the real intensity of the disease in domestic animals, and their epidemiologic role. The infectious context of SARS-CoV-2 in pets and whether there is any possibility of virus transmission to humans may lead to different conclusions regarding the Public Health authorities, depending on the implication of SARS-CoV-2 positive animals in the epidemiology of the disease for humans, it may dictate different Public Health Policies.

## Authors’ Contributions

LER and RCCM: Conceived and supervised the study and manuscript editing. OVC: Performed the research and drafted the manuscript. CKSF and DSR: Revised and finalized the manuscript for submission. All authors read and approved the final manuscript.
